# Immunomodulation by allograft endothelial cells

**DOI:** 10.3389/frtra.2025.1518772

**Published:** 2025-02-04

**Authors:** Sayantan Bose, Vicki Do, Chiara Testini, Suchita S. Jadhav, Nicolas Sailliet, Alvin T. Kho, Masaki Komatsu, Leo Boneschansker, Sek Won Kong, Johannes Wedel, David M. Briscoe

**Affiliations:** ^1^ Transplant Research Program, Boston Children’s Hospital, Boston, MA, United States; ^2^ Division of Nephrology, Boston Children’s Hospital, Boston, MA, United States; ^3^ The Department of Pediatrics, Boston Children’s Hospital, Boston, MA, United States; ^4^ The Department of Pediatrics, Harvard Medical School, Boston, MA, United States; ^5^ Computational Health Informatics Program, Boston Children’s Hospital, Boston, MA, United States

**Keywords:** endothelial cell, allograft (ALLO), immunoregulation, transplantation, graft survival

## Abstract

It is increasingly appreciated that the expression of immunoregulatory molecules within tumors have potential to shape a microenvironment that promotes local immunoevasion and immunoregulation. However, little is known about tissue-intrinsic immunomodulatory mechanisms following transplantation. We propose that differences in the phenotype of microvascular endothelial cells impact the alloantigenicity of the graft and its potential to promote immunoregulation following transplantation. We focus this review on the concept that graft-dependent immunoregulation may evolve post-transplantation, and that it is dependent on the phenotype of select subsets of intragraft endothelial cells. We also discuss evidence that long-term graft survival is critically dependent on adaptive interactions among immune cells and endothelial cells within the transplanted tissue microenvironment.

## Introduction

**Figure 1:**
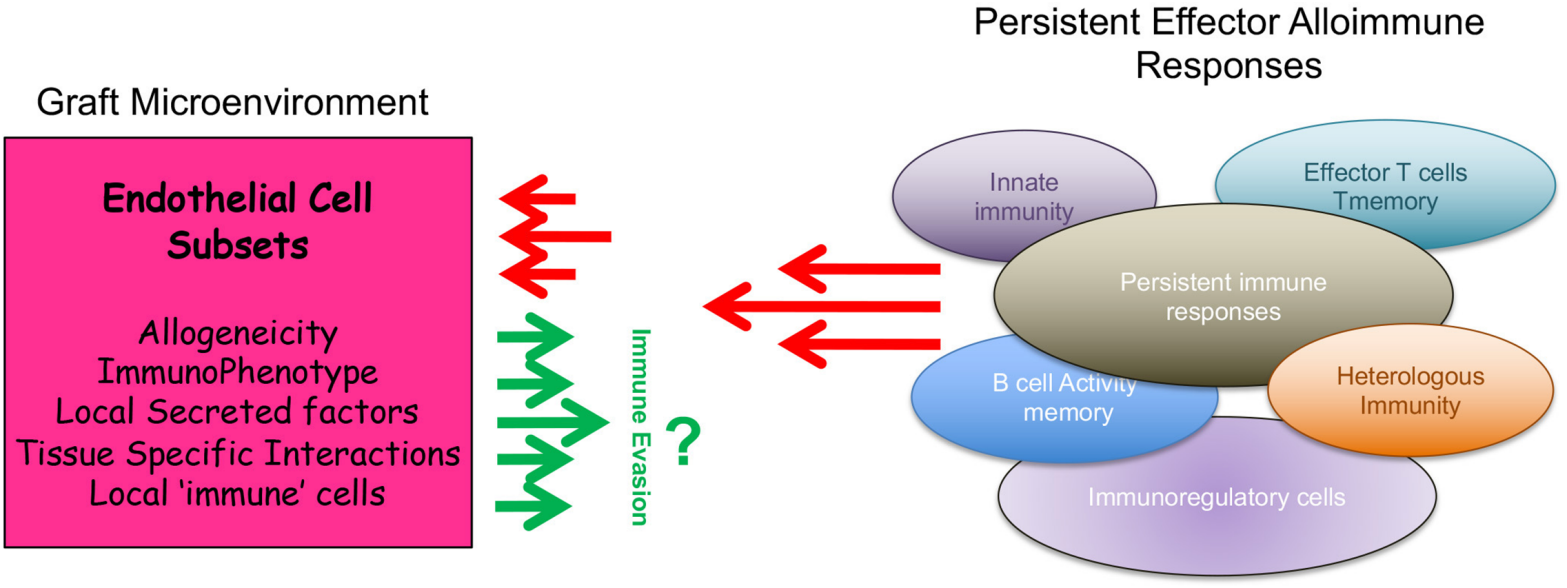
Cartoon illustration of how the phenotype of intragraft endothelial cell subsets regulate local alloimmunity to promote graft-dependent immunoregulation.

The development of rejection involves a marked inflammatory reaction characterized by effector T cell and B cell activation, an intense cellular and humoral response and an associated trafficking of alloreactive leukocytes into the allograft (1–3). Rejection is initiated by the recipient's immunological response to donor antigen, which is coordinated by CD4^+^ T cells that actively undergo expansion and differentiation into effectors and/or memory T cells (1, 4, 5). However, multiple pathways operate concurrently in order to control and regulate effector alloimmunity, and it is proposed that this process of immunoregulation can be a more potent component of the overall response (4–9). Indeed, the regulation of effector alloimmunity is complex, and classically involves several immune cell types (5, 9–15) including the expansion and function of CD4^+^Foxp3^+^ Tregulatory (Treg) cells (15–17). But importantly, it is also dependent on critical adaptive responses that occur within the graft itself (18–26). In this review, we focus our discussion on how obligate interactions among alloresponsive immune cells and multiple subsets of intragraft microvascular endothelial cells (EC) dictate the outcome of the rejection response. We postulate that the process of rejection is shaped by the immunogenic phenotype of select subsets of EC within a graft. Also, we suggest that it is possible to regulate the initiation of rejection through a process we have called graft-dependent immunoregulation (Figure 1).

## The phenotype of intragraft endothelial cells (EC) and graft-dependent immunoregulation

Multiple observations have established a functional role for graft vascular EC in the development of acute and chronic rejection (19, 22–24, 27–31). The expression of adhesion molecules and chemokines promote the recruitment of leukocytes into the graft, and the expression of MHC class I and II molecules, costimulatory molecules and cytokines promotes allogeneicity (25–27, 29, 32). These features enable EC to serve as semi-professional antigen presenting cells (APCs) and promote the activation of subsets of T cells (23, 27). Furthermore, the unique location of EC ensures obligate interactions with recipient immune cells. Since there are greater numbers of microvascular EC within a graft vs. professional APCs, there is a high likelihood that their interactions with infiltrating effector T cells dictate the nature of the reactivation response (23, 25, 33–36). Although controversial (37), direct interactions between CD4+ or CD8+ T cells and intragraft EC is sufficient to initiate and sustain allogeneic T cell activation as well as the rejection response (20, 25, 26, 38). Thus, the immunogenic phenotype of the EC has great relevance for the outcome of the local intragraft T cell immune response. Consistent with this interpretation, molecular profiling data and computational analysis in humans has confirmed that the state of activation, immunophenotype and allogeneicity of the graft microvasculature is associated with a microenvironment that is predictive of sustained rejection (39, 40). To date however bioinformatic approaches have not yet addressed the process of graft-dependent immunoregulation (24, 36).

Nevertheless, recent studies indicate that EC may express immunoregulatory molecules, including PD-L1, PD-L2, Tim-3, B7-H3, IDO and others, that are well established to modulate cell-mediated and alloimmune inflammatory reactions (24, 41–45). While little is known about EC-dependent immunoregulation following transplantation, several studies support a working model whereby the expression of select coinhibitory molecules on intragraft EC is both necessary and sufficient to support graft-dependent immunoregulation (18, 46–48).

Chalasani et al. (18) used a model in which fully MHC mismatched cardiac allografts were transplanted into splenectomized alymphoplastic (*aly/aly*) mice which lack secondary lymphoid tissues and accept allografts indefinitely. They found that the adoptive transfer of alloactivated T cells on day 2 post-transplantation resulted in graft failure, whereas transfer of the identical T cells on day 70 post-transplantation failed to precipitate rejection, all grafts surviving for >100 days. They also transferred alloactivated T cells into fully MHC mismatched C57BL/6 recipients of Balb/c cardiac allografts at similar time points following multidose treatment of recipients with CTLA4Ig and anti-CD154. Again, after a period of conditioning with costimulatory blockade (day 50 post-transplantation), adoptive transfer of alloreactive T cells failed to initiate acute rejection and all grafts survived long-term. Although these findings allowed for the interpretation that the graft itself has potential to determine the outcome of rejection, they did not identify the mechanism of graft-dependent immunoregulation in these studies. It is however most intriguing to consider that immunosuppressive agents and/or conditioning may induce select intragraft immunoevasive and/or immunomodulatory factors that shape these outcomes.

Riella *et al* (46). used a similar fully MHC mismatched C57BL/6 into Balb/c cardiac transplantation model and multi-dose CTLA4Ig treatment to prolong graft survival. These authors found that PD-L1 knockout grafts are rejected at an accelerated pace suggesting that local tissue expression is both necessary and sufficient to elicit graft-dependent immunoregulation. Consistent with this interpretation and the possibility that PD-L1 is functional on intragraft EC subsets, they also found accelerated rejection of allografts from bone-marrow chimeric mice in which PD-L1 is deficient in non-hematopoietic cells.

In another study, Koga et al. (48) used the minor MHC mismatched C57BL6 into B6.C-H2^bm12^ cardiac transplant model, which is known to result in a chronic insidious rejection process for >45 days (49, 50). They found that treatment with anti-PD-L1 resulted in accelerated rejection, characterized by marked inflammatory infiltrates, intragraft cytokine production and accelerated graft vascular arteriosclerosis vs. controls. In the same minor MHC mismatched B6.C-H2^bm12^ transplant model, Yang et al. used PD-L1 and PD-L2 knockout mice as donors and found that intragraft PD-L1, but not PD-L2, was functional to prolong graft survival (47). Furthermore, they also demonstrated that neither PD-L1 nor PD-L2 is functional in the regulation of the peripheral alloimmune response, conclusively discovering that intragraft PD-L1 is sufficient to elicit graft-dependent immunoregulation. Interestingly, it was also found that intragraft PD-L1 is functional to support early graft survival in a murine model of kidney transplantation, but these authors did not evaluate its expression on EC following immunosuppressive conditioning at later times post transplantation (51).

*In vitro* studies have demonstrated that select EC phenotypes suppress local alloimmune Teffector responses and/or augment the local activity of Tregs (24, 41, 42, 52). Thus, it is possible that select immunosuppressive therapeutics have potential to alter the phenotype of distinct subsets of intragraft EC to promote graft-dependent immunoregulation. Indeed, consistent with this hypothesis, pilot studies in our laboratory using a model of graft-dependent immunoregulation have revealed that EC within these grafts have a unique phenotype that includes regulation of the mTOR intracellular signaling pathways and the expression of multiple costimulatory, coinhibitory and immunoevasive molecules (53). Although mTOR inhibition can regulate coinhibitory gene expression by EC *in vitro* (42), understanding the mechanisms underlying immunoregulatory and immunoevasive gene expression *in vivo* will likely have significant implications for long-term transplant outcomes.

Overall, while little is known about EC- and graft-dependent immunoregulation following transplantation, the mechanism of tissue-dependent immunoevasion is an area of intense research in the tumor immunology field (24, 54–56). This process is functionally associated with coinhibitory molecule expression on EC. But it is not yet known if intrinsic heterogeneity within EC subsets, or differences in organ-specific production of immunoevasive and immunomodulatory genes impact the potential for graft-dependent immunoregulation.

## Heterogeneity in microvascular endothelial cell (EC) phenotypes both within and across different organs

Over the past 5–10 years, high-throughput single-cell RNA sequencing (scRNAseq) and spatial imaging technologies have brought new insights into the diversity and broad functions of microvascular EC subsets within tissues (36, 57). Several studies have determined that there is significant heterogeneity within microvascular EC subpopulations, and there are notable differences in EC phenotypes within different organs (36, 58–65). Although the significance of these differences has not yet been explored following transplantation, it appears that specialized EC subsets within different organs (notably, heart, lung and kidney) express unique gene signatures (36, 57, 60, 61, 65). Also, transcriptomic and epigenomic studies have demonstrated EC subset-specific differences in activation responses to pro-inflammatory stimuli (36, 59, 61, 63, 64, 66–71). This insight has brought new concepts to the transplantation field, for example that EC subsets within different organs respond with unique intracellular signals and gene expression signatures in the course of rejection and/or that EC subsets from different microvascular beds have potential to express immunoregulatory/immunoevasive gene signatures and thus resist Teffector mediated injury (36, 59, 63, 64, 66–74). Although previous studies indicated some heterogeneity in activation responses in human transplant biopsies by immunohistochemistry [for example (29, 75–77)], these new findings are suggestive of a paradigm whereby EC activation responses are not uniformal across subsets, but are rather unique to distinct subsets within each microvascular bed and/or across organs. The Valenzuela group reported that cultured human EC from heart, lung, liver, kidney and skin exhibited distinct inflammatory phenotypes at the mRNA level as well as in response to the pro-inflammatory cytokines TNFα and IL-1β (59). They speculated that diversity in activation phenotypes may contribute to differences in the injury response following transplantation. Moreover, tissue staining, microarray analysis and several other published scRNAseq studies of murine tissues indicate that there are at least 7 major EC subtypes within each organ microenvironment and that capillary EC are a most heterogeneous cell type with phenotypic differences both within and across different tissues (57, 61, 64, 65, 78).

**Figure 2:**
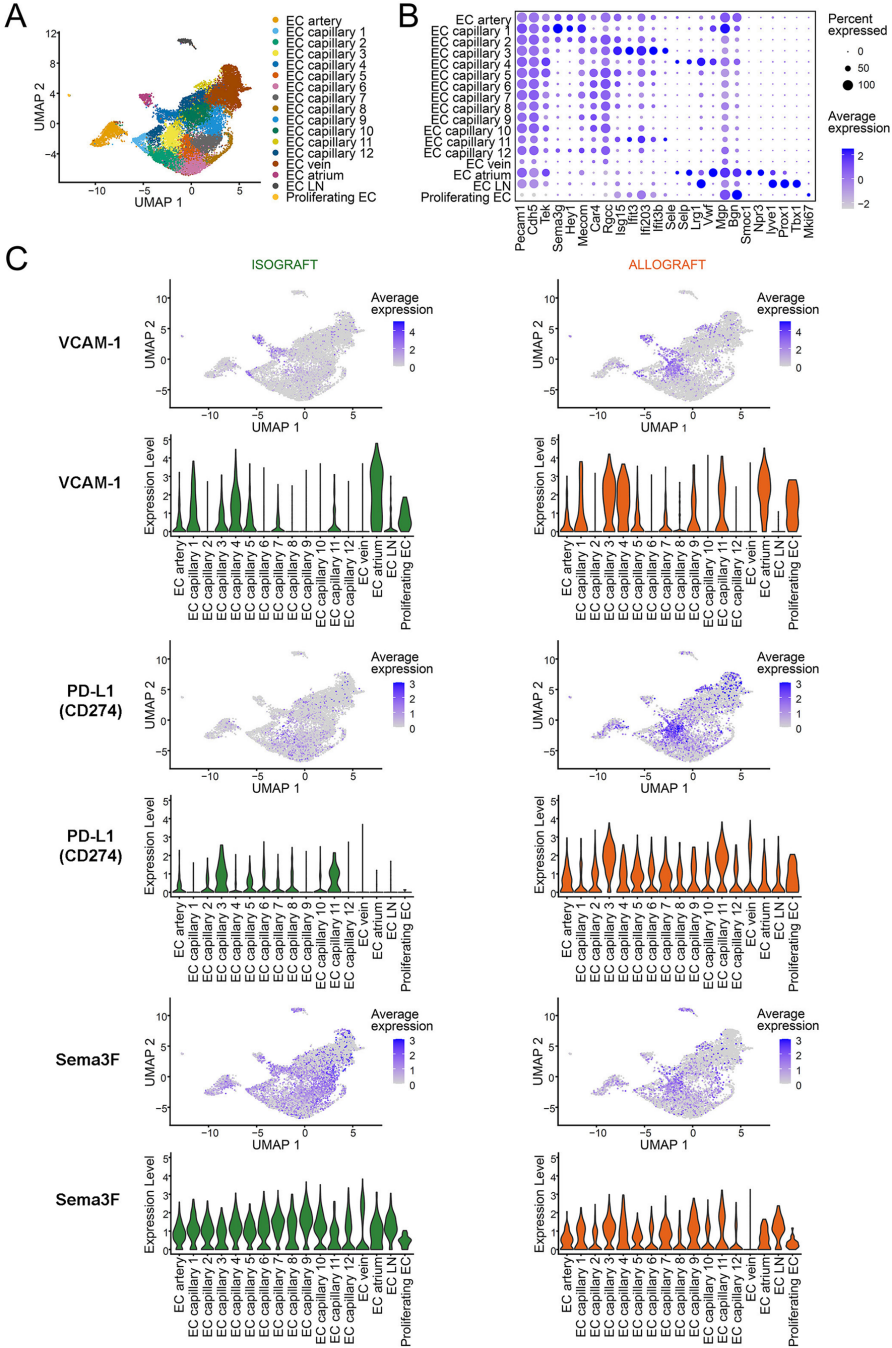
Intragraft microvascular endothelial cell heterogeneity from murine cardiac allo- and iso grafts. Single cell RNA-sequencing data from post-transplant day 5 murine cardiac isografts (Balb/c → Balb/c, *n* = 2) and allografts (Balb/c → C57BL6, *n* = 2) were downloaded from the NCBI GEO (accession number: GSE151048) (79). Seurat objects were generated from each sample, integrated using Harmony, and cluster resolution determined using the Clustree method (80–83). EC were identified using Pecam1 and Cdh5 expression and were clustered to identify subsets using selected EC annotation transcripts (57, 62, 80, 81). **(A)** UMAP scatter plot, color-coded for EC subclusters. EC were clustered based on established arterial, venous and capillary gene expression; a total of 12 capillary subsets are color coded. **(B)** Dot plot illustrating the transcripts used for EC subset annotation. The percent and level of expression of each transcript is illustrated by the size and color (blue) of each dot. **(C)** Feature and Violin plots of intragraft EC subsets isolated from isografts (left panels) or allografts (right panels) depicting select transcript expression of pro-inflammatory (VCAM-1), immunoregulatory (PD-L1) and immunoevasive (Sema3F) molecules. The color (blue dot) illustrates the level of expression of each gene in each Feature plot. Violin plots illustrate the relative level of expression of each gene in each EC subset.

**Figure 3:**
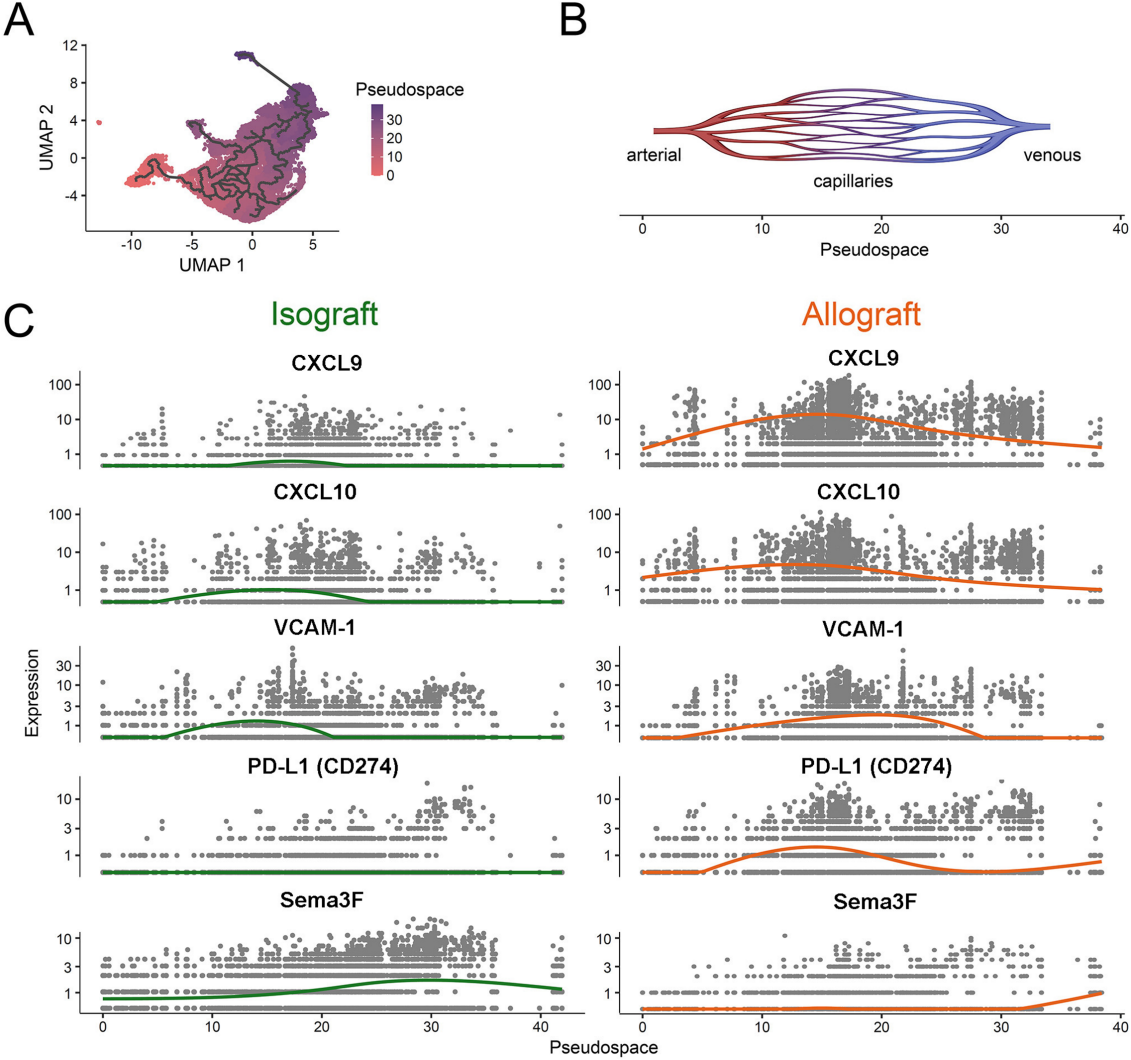
Pseudospatial expression of proinflammatory, immunoregulatory and immunoevasive transcripts in intragraft endothelial cells. A pseudotime estimation method was used to generate a pseudospatial resolution of single cell RNA-sequencing data from Figure 2 within the microvascular bed (84). The spatial trajectory starts in arterial microvasculature (as shown in Figure 2A), passes through capillaries and ends in venous EC subsets. **(A)** UMAP scatter plot color coded for the pseudospace. The trajectory is highlighted. **(B)** Schematic representation of the pseudospace within the microvasculature. **(C)** Scatter plots of isografts (left; green) and allografts (right; orange) depicting patterns of select proinflammatory (CXCL9, CXCL-10 and VCAM-1) or immunoregulatory/immunoevasive (PD-L1, Semaphorin3F) transcript expression over the pseudospace within each EC subset. Each line (green, isograft vs. orange, allograft) represents the average transcript expression within EC subsets along the pseudospace.

To highlight microvascular heterogeneity that evolves in established *in vivo* models of transplantation, we evaluated EC subset phenotypes and gene expression patterns in the initial post transplantation period by evaluating a recently published murine heart transplant scRNAseq dataset (79). As illustrated in Figure 2, we identify 12 distinct capillary EC subsets with marked phenotypic heterogeneity including distinct patterns of expression of pro-inflammatory [ICAM-1, VCAM-1 (29)], immunoregulatory [PD-L1 (46, 47)] and immunoevasive [Sema3F (24)] genes. We also compared gene expression patterns in C57BL/6 heart isografts and allografts harvested on day 5 post-transplantation (from C57BL/6 and Balb/c recipients respectively). As expected, the phenotype of EC subsets, expression levels and the distribution of well-established activation and immunoregulatory molecules differ within isografts and allografts (Figure 2). Furthermore, pseudotime trajectory analysis of pro-inflammatory chemokines (CXCL9, CXCL10), activation (VCAM-1), immunoregulatory (PD-L1) and immunoevasive (Sema3F) genes within this dataset identified distinct EC subset-specific patterns of expression of each gene within the microvasculature (Figure 3). Expression levels of each selected gene varied across the microvascular bed, with notable increases and decreases in expression in selected EC subsets (Figures 2, 3). These collective findings are consistent with the concept that distinct EC subsets within the transplanted tissue have potential to contribute to either pro-inflammation, immunoregulation and/or immunoevasion. Indeed, immunomodulation by select EC subsets can be influenced by the expression of coinhibitory molecules (23, 24, 36, 47, 55), cytokine-induced responses (24, 72, 73, 85), intracellular signaling responsiveness (24, 62, 77, 86) and the capacity to induce apoptosis in immune cells [for example, via FAS ligand (68)].

To add to the complexity of this biology, it has also been reported that EC within different organs express unique profiles of inflammatory or regulatory genes. For example, EC within the lung express gene signatures associated with immune activation, consistent with barrier organ biology, whereas EC derived from non-barrier organs such as the kidney and liver express genes associated with tissue-specific immune regulation (36). Jambusaria et al. (61) also found that EC within the brain, lung, and heart adapt to signatures from the surrounding tissue parenchyma; EC from the brain express genes associated with neuronal function and EC from the heart express genes associated with cardiac muscle development. Dumas et al. (60) identified 24 distinct EC subsets within the kidney, each with a unique transcriptional profile, for example with selective metabolic, IFN-responsive or antigen presentation phenotypes, and heterogeneity in EC phenotypes has been confirmed within transplanted kidneys (87). Thus, EC subsets within allografts may adopt profiles/phenotypes based on the cellular composition and/or immune events within the local intragraft microenvironment to support either chronic inflammation or immune regulation (88, 89).

### Mechanisms of graft-dependent immunoregulation

These new insights into the heterogeneity of EC within organs and tissues suggest that existing pro-inflammatory paradigms based on studies of single populations of EC are incomplete. For example, proinflammatory responses are not uniform across different EC subsets [artery, vein, capillary, see Figure 2 and (36)] or between EC from different organs (59). Furthermore, current paradigms suggest that pathological immune events within the intragraft microenvironment are shaped by local EC responsiveness to inflammation including local tissue hypoxia (19, 22, 90), cytokine production by infiltrating effector T cells (91) or resident immune cells (92), and tissue expression of local growth factors including VEGF-A (19, 31). Effector cytokines produced locally are well established to promote the activation of EC and support ongoing immune cell infiltration (19, 23, 24, 27). In contrast, during inflammation resolution multiple mediators produced by EC including pro-resolution lipids (93–95), anti-inflammatory cytokines (96) and/or neuronal guidance molecules (24, 97) promote immunoevasion to regulate leukocyte subset trafficking into tissues.

Physiological post-inflammatory mechanisms that resolve cell-mediated immune inflammation and sustain immune homeostasis are associated with the production and secretion of multiple families of molecules by EC, including neuronal guidance Netrins, Semaphorins and Slit family molecules that inhibit leukocyte trafficking into the tissue (24, 73, 93, 98–104). These immunoevasive proteins were originally described in the formation of the nervous system (105, 106), but they are expressed by multiple non-neuronal cell types, including EC, and their receptors are expressed on leukocyte subsets (97, 101, 103, 105). In this manner, neuronal guidance cues interact with immune cells and the response(s) elicit either chemoattractive or chemorepulsive signals (96). Thus, in the context of transplantation, intragraft expression of Netrins, Semaphorins and/or Slits have potential to influence the local phenotype of rejection response.

The Netrins are a family of secreted molecules that are structurally related to laminins and bind to uncoordinated 5 (UNC5) A-D, deleted in colorectal cancer (DCC), Neogenin, and the Down Syndrome cell adhesion molecule (DSCAM) receptors (98, 107–110). Immunoevasion elicited by Netrin-1 has been studied in immunity, and is dominantly attributed to interactions with the UNC5 family of receptors (98, 99, 111). The chemorepellent receptor UNC5B is expressed by peripheral blood mononuclear cells including neutrophils, T cells and monocytes, where it acts as an inhibitor of migration towards chemotactic stimuli (98, 109, 111) including inflammation that is associated with ischemia-reperfusion injury (99). In contrast, interactions between Netrin-1 and its neogenin receptor that is induced on activated CD4+ T cell subsets result in chemoattraction (98, 110). Thus, chemorepulsive UNC5 receptors or promigratory neogenin that are differentially expressed on CD4+ T cells may determine the immune response to local Netrin-1 expression within a tissue. Of note, neogenin can also bind to additional ligands involved in the regulation of T cell activation (112), indicating that its expression on leukocytes may dictate the relative immunomodulatory function of local EC-derived Netrin-1 in the course of cell-mediated immune inflammation and/or rejection.

The semaphorins (Semas) are immunomodulatory proteins that were also discovered as neuronal guidance cues (97, 113–117). Semas consist of eight families, most of which are membrane bound, and vertebrate members (Sema families 3–7) are reported to function in the immune response (114–116, 118). The Sema3 family members (Sema3A-G) are soluble secreted proteins, and some (for example Sema3F and 3G) are expressed at high levels by EC (65). These proteins bind to neuropilin (NRP) -1 and NRP-2 that are expressed by T cell subsets (73, 118–121). NRP-1 is a marker of activated CD4+ Foxp3+ Treg cells (118, 122, 123), but recent studies have also identified its expression on antigen-activated and exhausted CD8^+^ T cells (119, 120). We (73) and others (118, 119) have observed that the interaction between Sema3 and Sema4 proteins with NRP1/2 results in an inhibition of PI-3K/Akt/mTOR signalling as well as cytoskeletal collapse and reduced migration in multiple cells types including lymphocytes. Knockout of NRP-1 on lymphocytes is associated with enhanced migration and effector function of CD4+ and CD8+ T cells (119). In contrast, the stimulation of NRP-1 on Tregs enhances stability and function (118). Thus, EC expression of Sema3 family proteins (see Figures 2, 3) is likely to have potent implications for both the migration and activation of NRP-expressing effector and regulatory cells within allografts.

The Slit family of proteins are also immunoevasive neuronal guidance cues that are expressed by EC at lower levels (103, 104), but little is known about their tissue expression or biology in the resolution of cell-mediated immunity. Nevertheless, Slit-2 has been shown to inhibit the migration of leukocytes in response to chemokines via interactions with the Roundabout (Robo) family of receptors that are expressed on leukocytes (104, 124). Expression is also reported to protect and inhibit neutrophil-induced chemotaxis (96, 104) as well as ischemia-reperfusion injury (102), but to our knowledge their biology has not yet been explored following transplantation.

**Figure 4:**
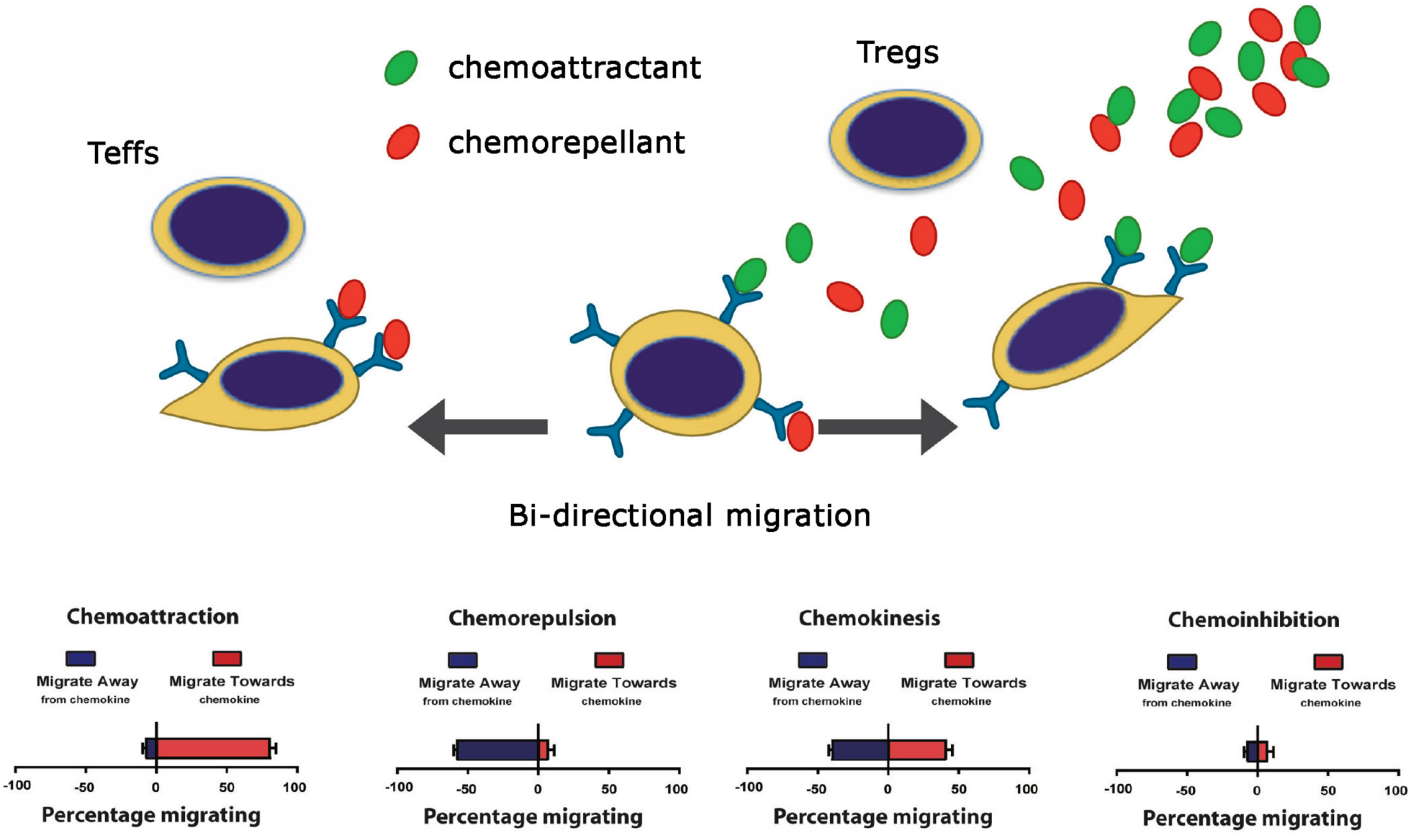
Cartoon illustration of the four patterns of leukocyte migration (96).

Since little is known about the biology of immunoevasion, in previous studies, we developed an *in vitro* platform to evaluate attraction and inhibition of leukocyte migration simultaneously. In this manner, it was possible to evaluate the effects of migratory guidance cues on bidirectional leukocyte trafficking patterns (96). We discovered that migration and leukocyte trafficking is more complex than previously described (125–129), as the migratory response (or lack of) does not simply relate to chemoattraction. Rather, migratory responses occur in at least four distinct patterns, called chemoattraction, chemorepulsion, chemoinhibition, and chemokinesis (96), as illustrated in Figure 4.

Tissue-dependent immunoevasion may be elicited in part through the process of chemorepulsion that results in migration in the opposite direction to chemoattraction (96). Furthermore, a chemoinhibitory stimulus [such as a response to Slit family molecules (96, 102, 103)] reduces migration in random directions to a guidance cue. Importantly, some molecules [for example Netrin-1 (98, 100, 111)] promotes a bidirectional migratory response with potential to elicit both chemorepulsion and chemoattraction depending upon the relative expression of its receptor(s) on each leukocyte subset(s). Also, members of the semaphorin family, including endothelial Sema3F [(73) and Figures 2, 3] or SDF-1/CXCL12 (96) elicits chemorepulsive and/or dispersive signals via receptors expressed on distinct subsets of immune cells, including CD4+ T cells (122, 130). Collectively, these studies indicate that combinations of guidance molecules expressed by EC subsets may serve to inhibit leukocyte migration and extravasation into allografts but the process of chemoinhibition and/or chemorepulsion is also dependent on the relative levels of chemotactic receptors expressed on individual infiltrating immune cells.

Another important consideration is whether the characteristics of resident immune cells within the graft alter the EC subset phenotype, or whether the EC subset phenotype regulates the characteristics of the local intragraft immune infiltrate. The Lakkis group demonstrated that initial effector cell infiltration into an allograft requires recognition of alloantigen, likely expressed on locally activated and MHC-expressing EC subsets (26). Furthermore, they found that the differentiation of effectors into pathological T resident memory cells (T_RM_) requires antigen presentation as well as cytokine-induced activation (88, 92). Abou-Daya et al. (88) found that recipient graft infiltrating effector T cells acquire a T_RM_ phenotype and that these cells sustain their localization within an allograft where they produce effector proinflammatory cytokines (88). Although Tieu et al. later demonstrated that the maintenance of T_RM_ within the graft was dependent on antigen presentation by intragraft dendritic cells (92), the role of interacting EC subsets in the persistence of T_RM_ localization was not evaluated.

In addition, the function of EC subsets in the recruitment of Tregs into an allograft is poorly understood, but it is also likely to involve the recognition of antigen as well as local activation responses (131, 132). In select transplant models and/or following immunosuppressive conditioning, perivascular aggregates of Tregs are recruited into the graft where they localize into Treg-rich Organized Lymphoid Structures (TOLS) that are reported to promote immunoregulation and support long-term graft survival (89, 133–135). TOLS-containing allografts elicit an immunoregulatory response following retransplantation into fully MHC mismatched recipients (133) and early depletion of Foxp3+ Tregs within TOLS results in allograft rejection (134, 136). These findings confirm a role for TOLS in graft-dependent immunoregulation. Nevertheless, it is not known if the presence of intragraft TOLS is associated with changes in the phenotype of local EC within a graft, or whether local EC subsets adapt and express immunomodulatory genes (e.g., Sema proteins) that support Treg localization and thus graft-dependent immunoregulation. Furthermore, as discussed above, EC- and graft-dependent mechanisms of immunoregulation may occur in the absence of CD4+ T regulatory cell recruitment or TOLS development, for example following a period of immunosuppression after transplantation in recipients treated with costimulatory blockade (18, 46). Thus, the development of EC phenotypes that support graft-dependent immunoregulation likely involves an independent cell-intrinsic mechanism and/or a modulatory signaling response(s) within the local tissue microenvironment (24, 36, 42).

## Therapeutic implications

Cell-intrinsic mTOR signaling in EC is well established to play a central role in EC activation responses (86, 137–141), and its biology in EC is implicated in a large number of human inflammatory diseases (86, 142, 143). Targeting mTOR in EC with pharmacological mTOR inhibitors, even at low concentrations (144), inhibits EC activation (30, 86, 138) and has marked effects on the augmentation of coinhibitory PD-L1 and PD-L2 expression (42). This response has been reported to be associated with graft-dependent immunoregulation (as discussed above) and to enhance local immunoregulation in part via the augmentation of Treg function (42, 52, 145). Thus, treatment with mTOR inhibitors [event at low doses (144)] may target the graft EC to promote immunomodulation independent of its effects on the peripheral alloimmune response (86).

DEPTOR is a potent cell-intrinsic regulator of mTOR (146) that is expressed at variable levels within EC subsets *in vitro* and *in vivo* (77). It was originally identified to modulate mTOR signaling activity via its dominant ability to bind and inhibit mTORC1 complex assembly (146–148), but it also regulates the MAPK and STAT signaling pathways in EC (77, 146). Interestingly, rapamycin augments DEPTOR expression (147), suggesting another mechanism whereby it may be therapeutic to target EC activation. siRNA knockdown of DEPTOR in EC has a striking effect on the induction of activation gene expression signatures with up to a 1,000-fold increase in the expression of select chemokines (77). In addition, a recently published study indicated that knockout of EC DEPTOR has similar biological consequences *in vivo* (149). Since DEPTOR is a potent regulator of mTOR, its biology in EC is thus directly linked to intragraft inflammation and immunoregulation. Overall, these findings suggest that cell intrinsic modulation of mTOR signaling is both necessary and sufficient for EC-dependent immunoregulation. They also suggest that therapeutics that inhibit mTOR activity and/or sustain the expression of cell-intrinsic DEPTOR will be of great significance to support the development of graft-dependent immunoregulation.

## Summary and future outlook

The understanding of tissue-dependent immunoregulation is driven by studies in the tumor literature, and little is known about underlying mechanisms within allografts. In this review, we discuss the literature demonstrating that it is possible to augment graft-dependent immunoregulation following a period of immune conditioning. We also review literature showing that the inhibition of mTOR signaling and/or cell intrinsic modulators of proinflammatory signals in EC have potential to induce an immunomodulatory phenotype. However, there is a need to evaluate and study the heterogeneity in phenotypes within the allograft microvasculature, differences in EC phenotypes and responses across different organs and changes that occur following transplantation. In this manner, it may be possible to uncover unique signals that drive EC phenotypes that are associated within immunomodulation and long-term graft survival. Future research studies may also identify mechanisms whereby EC adopt microenvironmental cues to promote either pro-inflammation or immunoregulation. Deciphering fundamental mechanisms underlying how different EC subsets within different organs adapt in order to regulate and modulate the local immune response will have significant clinical implications in the field. We predict a future whereby different graft-targeted therapeutics will be used following organ transplantation to sustain the induction of local genes that promote immunomodulation. Another potential future outlook relates to the monitoring of grafts for immunoregulatory gene expression as a determinant of long-term outcome.
